# Functional changes in Becker muscular dystrophy: implications for clinical trials in dystrophinopathies

**DOI:** 10.1038/srep32439

**Published:** 2016-09-01

**Authors:** Luca Bello, Paola Campadello, Andrea Barp, Marina Fanin, Claudio Semplicini, Gianni Sorarù, Luca Caumo, Chiara Calore, Corrado Angelini, Elena Pegoraro

**Affiliations:** 1 Department of Neurosciences, University of Padova, Padova, Italy; 2 Department of Cardiac, Thoracic and Vascular Sciences, Cardiology Clinic, University of Padova, Padova, Italy

## Abstract

We performed a 1-year longitudinal study of Six Minute Walk Test (6MWT), North Star Ambulatory Assessment (NSAA), and timed function tests in Becker muscular dystrophy (BMD). Skeletal muscle dystrophin was quantified by immunoblot. We grouped deletions ending on exon 45 (“del 45-x”, n = 28) or 51 (“del x-51”, n = 10); isolated exon 48 deletion (“del 48”, n = 10); and other mutations (n = 21). Only patients in the “del 45-x” or “other” groups became non-ambulatory (n = 5, log-rank p = n.s.) or unable to run (n = 22, p < 0.001). All measures correlated positively with dystrophin quantity and negatively with age, and were significantly more impaired in the “del 45-x” and “other” groups. After one year, NSAA score decreased significantly (−0.9 ± 1.6, p < 0.001); in the “del 45-x” group, both NSAA (−1.3 ± 1.7, p = 0.001) and 6MWT (−12 ± 31 m, p = 0.059) decreased. We conclude that patients with “del x-51” or “del 48” mutations have mild or asymptomatic BMD, while “del 45-x” mutations cause comparatively severe weakness, and functional deterioration in 1 year. Furthermore, exon 51 skipping could be more effective than exon 45 skipping in Duchenne muscular dystrophy.

Becker muscular dystrophy (BMD) is an X-linked disorder caused by non-truncating *DMD* mutations, consisting approximately of 70% large deletions, 15% duplications, and 15% small mutations, leading to altered, but detectable dystrophin expression in muscle fibers^1^,^2^.

The “typical” presentation of BMD features a juvenile onset of muscle wasting and weakness, predominant at the thigh extensors and pelvic girdle, calf hypertrophy, gradual progression leading to loss of motor function over years or decades, and frequent dilated cardiomyopathy, not proportional in severity to muscle involvement^2^. According to a classic definition, BMD patients lose ambulation after the age of 16 years, while in the severe allelic disorder Duchenne muscular dystrophy (DMD), caused by truncating mutation and absent dystrophin, ambulation is lost by 13^2^. However, BMD also includes patients with calf hypertrophy and/or elevated creatine kinase, but virtually no muscle weakness^3^.

Different BMD deletions affect the properties of the resulting dystrophin protein: the loss of functionally critical N- or C-terminal domains may result in DMD-like phenotypes^4^,^5^,^6^, while the consequences of deletions in the dystrophin rod domain depend on structural “phase” between spectrin repeats and hinge regions^7^. Deletions including in-frame exons in the proximal rod domain^3^, or the hinge 3 domain encoded by exons 50–51^8^,^9^, have been associated to mild phenotypes; while deletions situated in the exon 45–53 mutational hotspot^10^, but not including exons 50–51, usually cause “typical” BMD^11^,^12^,^13^. Moreover, linear or threshold correlations between dystrophin quantity in skeletal muscle tissue and BMD severity have been described^3^,^9^,^13^,^14^,^15^. Interest has been rekindled in this field since splice-modulating antisense oligonucleotides (AONs) have been introduced, aiming to convert the DMD phenotype into BMD with the exon skipping approach^16^,^17^.

The longitudinal description of validated, clinically meaningful outcome measures is needed for the design of BMD clinical trials. Unlike DMD^18^,^19^,^20^,^21^,^22^, there is scarce data in BMD about standardized functional measures such as the Six Minute Walk Test (6MWT)^23^, the North Star Ambulatory Assessment (NSAA)^24^,^25^,^26^, and Timed Function Tests (TFTs: run/walk 10 m, rise from the floor, climb four standard steps). These were evaluated at baseline and after one year in a population of BMD patients referring to the Neurology Clinic at the University of Padova, who were also characterized at the level of their *DMD* gene mutations (in all patients) and skeletal muscle dystrophin content (when available). We aimed to explore if these measures are feasible and clinically meaningful in BMD as they are in DMD, and to refine the description of the natural history of relevant BMD mutational subgroups.

## Methods

### Ethics statement

All evaluations involving patients and experiments involving muscle tissue samples were performed in accordance with relevant guidelines and regulations, and were approved by the Padova Ethics Committee for Clinical Experimentation. All patients, or their legal guardians, provided their written informed consent to study procedures.

### Inclusion criteria

We selected male BMD patients with (a) an in-frame *DMD* mutation; or (b) muscle immunoblot or immunofluorescence showing non-absent dystrophin, and any *DMD* mutation.

### Dystrophin quantification

Protein samples from diagnostic biopsies were separated by SDS-PAGE on 3–8% gradient Tris-glycine gel, and transferred for 5 hours onto a nitrocellulose membrane. We used a primary monoclonal antibody against the dystrophin C-terminus. Visualization on X-ray films was performed by ECL-chemiluminescence (Amersham). Adult male control samples were loaded in the same gel to determine relative abundance. Dystrophin quantity was determined by densitometry of dystrophin bands (ImageJ software), normalized to myosin bands in the post-transfer Coomassie blue stained gels, with subtraction of background.

### DNA analyses

Molecular analyses were performed in the context of diagnostic testing and included multiplex ligation-dependent probe amplification (MLPA) and genomic sequence of *DMD* exons and flanking regions. Single-exon deletions identified by MLPA were confirmed by PCR, using primers which did not overlap MLPA probe binding regions.

### Functional evaluations

6MWT, NSAA, and TFTs (run/walk 10 m, rise from floor, climb 4 stairs) were performed as described^21^,^24^,^25^,^26^ by trained neuromuscular physicians (LB, AB, CS), at baseline and after 12 ± 1 months. To ensure inter-rater reliability, a main evaluator (AB) participated in all study visits. In the 6MWT, patients were instructed to walk up and down a corridor, turning around cones positioned at 25 m distance from each other at each end of the corridor, at a self-paced velocity, with the aim of covering the longest possible distance (without running) in six minutes. Distance was measured as number of turns x 25 m, + distance from the last cone at 6 minutes. The NSAA consists of 17 items related to everyday motor activities: standing, walking, standing on one leg (left and right), standing up from a chair, climbing and descending a box step with each foot, raising from the floor, sitting up from supine, lifting one’s head from supine, standing on heels, jumping on both feet, hopping on each foot, and running. Each item was scored as 2 (able to perform normally), 1 (able to perform independently with compensatory strategies), or 0 (unable to perform independently), yielding possible scores of 0–34. In the “run/walk 10 m” test, patients were instructed to “run or walk as fast as they safely can” down a 10 m stretch, time taken was measured, and velocity was calculated as 10 m/time in s. “Rise from floor” velocity was calculated as 1/time in s taken to stand up from a sitting position on the floor with legs outstretched. “Climb stair” velocity is calculated as 4/time in s taken to climb 4 standard stairs. Patients unable to perform tasks were scored “zero” for baseline analyses, and excluded from longitudinal analyses. Loss of ambulation (LoA) was defined as continuous wheelchair use, and loss of the ability to run (LoR) as inability to accelerate and lift both feet off the ground; these “disease milestones” were evaluated retrospectively.

### Mutation groups

We grouped *DMD* deletions predicted to result at transcript level from skipping of exons 45 and 51: respectively, del 45–47, 45–48, 45–49, and 45–55 (“del 45-x”), and del 34–51, 45–51, 48–51, and 50–51 (“del x-51”). There was a double rationale to this grouping criterion: first, “del 45-x” BMD mutations have been described in the literature to be associated with a more severe phenotype than “del x-51”^8^,^9^,^10^,^11^,^12^; second, functional measure changes in these groups may be taken as a model of successful exon skipping in corresponding DMD populations^9^. The relatively frequent “del 48” was grouped separately, and all remaining patients were included in a miscellaneaous “other” mutation group.

### Cardiomyopathy

While an in-depth discussion of cardiomyopathy is beyond the purposes of this paper, in order to better describe the clinical characteristics of the studied cohort, we assessed the frequency of dilated cardiomyopathy (DCM) in the overall cohort and in defined mutation groups. We defined DCM echocardiographically as left ventricular ejection fraction (LVEF) <50%, and/or increased left ventricular diastolic volume (LVEDV >70 ml/m^2^), including patients with previous symptoms/signs of heart failure that in most severe cases needed cardiac transplantation. We also gathered data about LVEF and LVEDV in a time window of one year before or after baseline evaluations, when available, to assess the impact of cardiac dysfunction on motor outcome measures, and especially the 6MWT, which is most influenced by fatigability and exercise tolerance, and thus most likely modified by cardiac impairment.

### Statistical analyses

Age between groups was compared by Student’s t, and dystrophin by Mann-Whitney U test. Age at LoA/LoR was compared by log-rank test, and correlated to dystrophin by Cox regression. Correlations were assessed by Spearman’s rank coefficient. Baseline functional differences were tested by Kruskal-Wallis rank sum test. ANCOVA was used to assess independent effects of age and mutation groups on baseline functional measures, as well as concurrent effects of muscle weakness (NSAA) and cardiac dysfunction (dichotomic DCM vs. no DCM variable, and quantitative LVEF and LVEDV variables) on 6MWT distance. Significance of functional changes was tested by Friedman rank sum test. Dystrophin quantity was treated as a continuous variable for correlations, and categorized (0–33%, 34–66%, and 67–100%) for descriptive statistics. Statistical significance was set at p < 0.05. Analyses were performed with R (v3.2.1), and power calculations with PS v3.0.4328 (paired t-test).

## Results

### Patients

We recruited 69 BMD patients, aged 6 to 69 years, from 61 unrelated families. Familial cases included one sibship of three and four sibships of two brothers, one uncle-nephew pair, and one grandfather-grandson pair (Table 1).

### Mutations

Fifty-six/69 (81%) patients harboured large deletions. Two (3%) harboured duplications. In-frame microdeletions were identified in one patient (c.10099_10101delGAA, p.Glu3386del) and three brothers (c.676_678delAAG, p.Lys226del) (Table 1). Two brothers had a frame-shifting microdeletion (c.10507_10508delAG, p.Lys3505AlaFsX8) in exon 74; a muscle biopsy showed reduced, but widespread immunolabeling with an anti-rod antibody. One patient had a missense mutation (c.478A > C, p.Thr160Pro), and two brothers had a synonymous mutation (c.4299G > T, p.Gly1433Gly) predicted *in silico* to disrupt an exon splicing enhancer (ESE)^27^. RNA studies were unavailable, but the variant was not found in the dbSNP, 1000 Genomes, and ExAC databases, the remaining gDNA sequence was normal, and immunoblot showed reduced (10–30%) dystrophin with a 400 kDa molecular weight. Two patients harboured nonsense mutations: c.4980G > A, p.Trp1660* in exon 35, with 29%, 400 kDa dystrophin; and c.3843G > A, p.Trp1281* in exon 28, with 17%, 400 kDa dystrophin. Grouped by mutation (see Methods), there were 28 “del 45-x” patients, 10 “del 48”, 10 “del x-51”, and 21 “other”. Average age in the “del 48” and “del x-51” groups was younger than “del 45-x” group (p = 0.08 and 0.02, respectively).

### Dystrophin quantity

Average dystrophin quantity was 59.5 ± 31.3% of control. Dystrophin was more abundant in the “del x-51” than “del 45-x” group (p = 0.03). Deletions 45–55, 10–25, 10–29, and 11–30 showed dystrophin quantities similar to control (Table 1).

**Figure 1:**
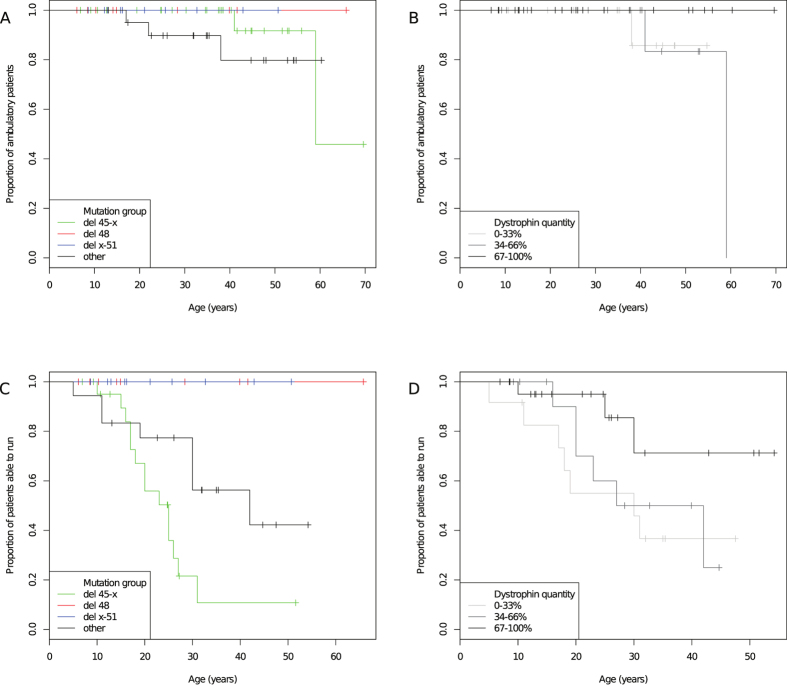
Kaplan-Meier plots of loss of ability to walk and run. Age at LoA is represented, grouped by (**A**) mutation group (“del 45-x”, “del 48”, “del x-51”, and “other”), and (**B**) dystrophin quantity (0–33%, 34–66%, 67–100%). The proportion of patients able to run at increasing ages are represented by (**C**) mutation group, and (**D**) dystrophin quantity. Censored patients (able to walk or run at last follow-up) are indicated by crosses.

*LoA.* At baseline, 3/69 patients were full time wheelchair users: one 60-year-old (del 45–48, LoA at 59); one 47-year-old (del 45–47, LoA at 41); and one 18-year-old (in-frame microdeletion, LoA at 17). Two patients lost ambulation during follow-up: one 22-year-old (out-of-frame microdeletion); and one 38-year-old (missense mutation). LoA only occurred in the “del 45-x” or “other” groups (Fig. 1, panel A), although the log-rank test was not statistically significant. Grouping by dystrophin quantity, no LoA events were observed in the high (67–100%) dystrophin category (Fig. 1, panel B), although the Cox regression analysis did not show a significant effect of % dystrophin as a predictor of LoA, possibly because of the small number of events.

*LoR.* Similarly, only patients in the “del 45-x” (14/28, 50%) or “other” (11/21, 52%) groups stopped running. Nine could not date LoR precisely, and were excluded from time-to-event analyses. Median age at LoR was 31 overall, 95% confidence interval (CI) 26 - undetermined, and 26 in the “del 45-x” group, 95% CI 17–27. Age at LoR differed significantly between mutations (log-rank p < 0.001 with 3 degrees of freedom, Fig. 1, panel C), and was significantly associated to dystrophin quantity (Hazard Ratio 0.98, 95% CI 0.90~0.99, p = 0.03, Fig. 1, panel D).

### Baseline 6MWT

One patient refused to perform the 6MWT because of recent trauma. Four were assigned a “zero” distance: three were non-ambulatory and one could only take a few steps with a walking frame. Average 6MWT distance was 391 ± 155 m (414 ± 123 m excluding “zero” values) (Table 2). All tests were completed with no falls, dyspnea, dizziness, palpitations, arrhythmia, or other cardiac or respiratory symptoms.

### Baseline NSAA

NSAA was scored in 68/69 patients (one missing score because of recent trauma) and averaged 25.3 ± 10.8, with a range of 2–34 and a right-skewed distribution (median 32.5) (Table 2). Twenty-eight patients scored 34/34. The minimum score was 2/34 (all patients were able to lift their head from supine, scoring 2 points in the corresponding item).

### Baseline TFTs

One patient refused to perform the 10 m walk/run because of recent trauma, and one could not perform TFTs because of a logistic issue. Due to disease progression, 4/67 patients (6%) were unable to walk 10 m, 18/68 (26%) were unable to rise from the floor, and 9/68 (13%) were unable to climb 4 standard steps (“zero” velocities) (Table 2).

### Baseline correlations

**Figure 2:**
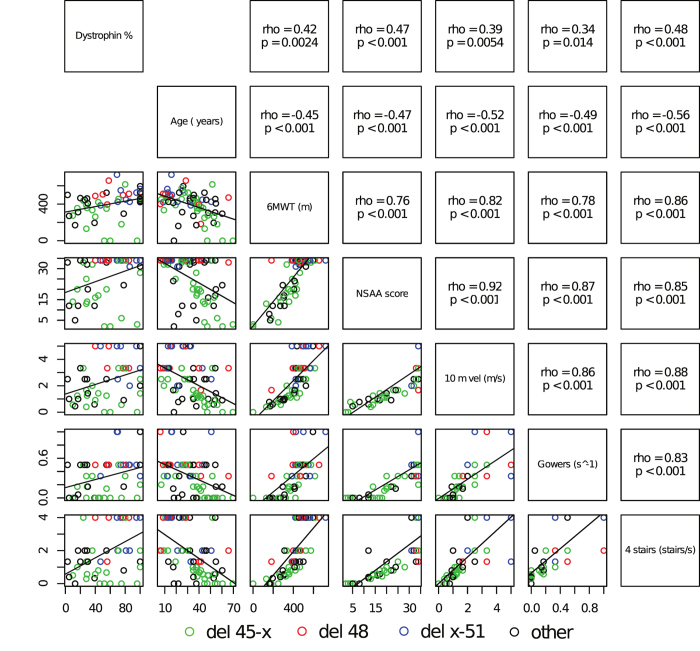
Correlation matrix of dystrophin quantity, age, and functional measures. Panels in the diagonal indicate measures represented on corresponding columns and rows. Upper panels show correlation parameters (Spearman’s ρ and corresponding p-value) between measures on corresponding the row and column, while lower panels show scatter plots, with data points color-coded for mutation group, and corresponding regression lines. Correlations between dystrophin quantity and age is not shown, because age represented here is age at study procedures, and not age at biopsy (which may be years or decades earlier).

All functional measures showed a moderate positive correlation with dystrophin quantity, a moderate negative correlation with age, and strong positive inter-correlations. Due to NSAA “ceiling effect”, correlations between NSAA and other parameters appeared more linear in more severe groups (“del 45-x”, “other”), although still significant in the overall BMD cohort (Fig. 2).

### Baseline functional differences between mutations

All functional measures differed significantly between the four mutation groups at baseline (Kruskal-Wallis rank sum test p = 0.02 for 6MWT, and p < 0.001 for NSAA and TFTs) (Table 2). Compared to “del 45-x”, “del 48” and “del x-51” patients covered on average respectively 112 and 150 m more in the 6MWT. Furthermore, while NSAA scores were simmetrically distributed in the “del 45-x group” (mean ± SD 20.9 ± 11.1, median 20), 90% of “del 48” and “del x-51” patients scored 34/34. The latter were also faster in all TFTs.

### Independent effects of mutation and age on functional measures

As all functional measures were negatively correlated with age, and patients in the “del 48” and “del x-51” mutation groups, who presented milder or no functional impairment, were on average younger than those in the “del 45-x” group, we asked if functional differences between these two group could be wholly attributed to age, or if there was an independent effect of mutations. We performed analyses of covariance (ANCOVA) with baseline NSAA and 6MWT as dependent variables, and mutation group and age as independent variables. This showed that mutation group and age have significant, independent effects on NSAA (p < 0.001 for both mutation and age) and 6MWT (p = 0.004 for mutation and p < 0.001 for age). The different slope in NSAA and 6MWT values, plotted against age, in different mutation groups, is evident in Supplementary Fig. 1, where NSAA and 6MWT values in “del 48” and “del x-51” patients appear stable, while they decrease with age in “del 45-x” patients.

### One-year functional changes

**Figure 3:**
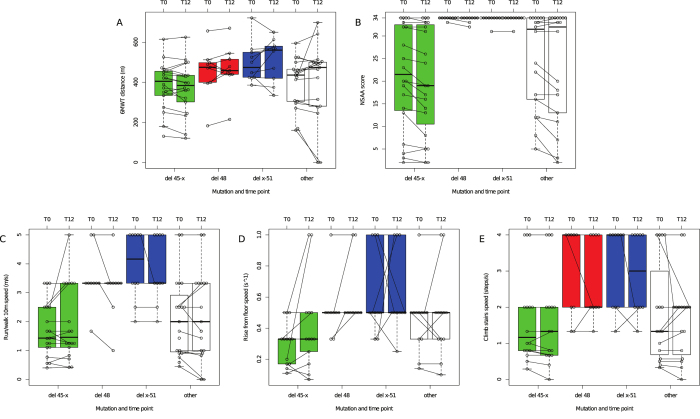
Functional changes after 1 year in different BMD mutation groups. Box plots showing baseline and 1-year values of (**A**) 6MWT distance, (**B**) NSAA score, (**C**) 10 m run/walk velocity, (**D**) rise from floor velocity, and (**E**) climb 4 standard steps velocity. Boxes are color-coded for mutation group, and trajectories of each individual patient are illustrated by dots connected by segments. Thick lines represent median values.

**Figure 4:**
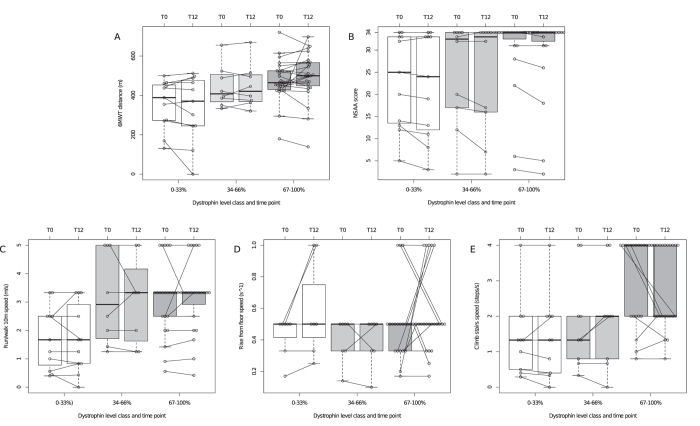
Functional changes after 1 year in different dystrophin quantity level groups. Box plots showing baseline and 1-year values of (**A**) 6MWT distance, (**B**) NSAA score, (**C**) 10 m run/walk velocity, (**D**) rise from floor velocity, and (**E**) climb 4 standard steps velocity. Boxes are color-coded for dystrophin levels of 0–33%, 34–66%, and 67–100% relative to control, and trajectories of each individual patient are illustrated by dots connected by segments. Thick lines represent median values.

One-year 6MWT was stable overall (+3 ± 66 m). Conversely, NSAA decreased significantly (−0.9 ± 1.6, p < 0.001) (Table 2). TFTs were stable. Two patients who lost ambulation had covered 161 and 170 m at baseline 6MWT. No patient lost the ability to rise from the floor, while two (age 47, del 45–47; and age 53, c.676_678delAAG) lost the ability to climb steps. Functional changes within groups (Fig. 3) showed that patients in the “del 45-x” group lost −12 ± 31 m at the 6MWT (p = 0.059), and −1.3 ± 1.7 NSAA points (p = 0.001). We did not observe significant TFT velocity changes. There was a trend of correlation between dystrophin and 1-year 6MWT (p = 0.055) and NSAA changes (p = 0.09), but not TFT changes (Table 3, Fig. 4).

### Power calculation for a hypothetical BMD clinical trial

**Figure 5:**
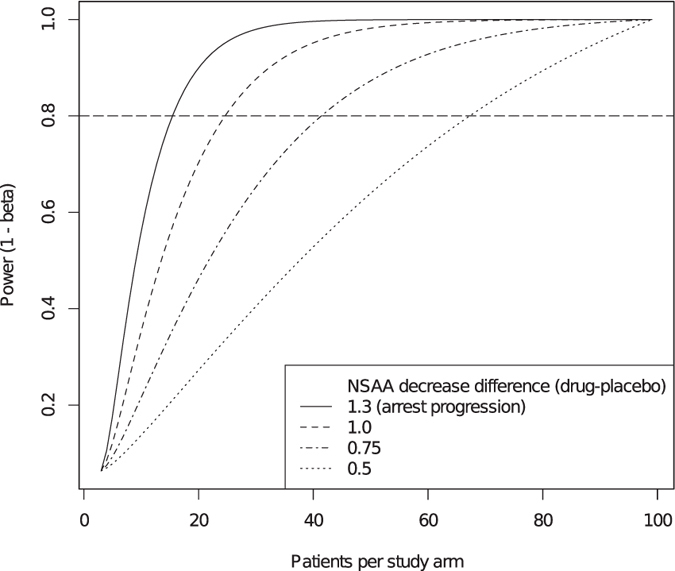
Power calculation for a hypothetical 1-year trial in typical BMD, using NSAA as an outcome measure. This power calculation assumes selection of BMD patients with a typical BMD phenotype based on functional or genetic criteria, and exclusion of patients with mild/asymptomatic or DMD-like phenotypes; an accepted type I error rate of α = 0.05, and a required statistical power (1−β) = 0.8, and a blinded placebo-controlled design. The number of patients per study arm required for adequate power increases, as the hypothetical difference in 1-year NSAA change between treatment and placebo decreases.

We asked how many BMD patients would be needed to demonstrate effectiveness of a 1-year treatment in slowing NSAA decrease against placebo. As NSAA decrease was significant in the “del 45-x” group, we used data from this group, which we deem to be representative of a “typical” BMD population (excluding asymptomatic and DMD-like patients). Assuming α = 0.05 and power = 0.8, identifying the effect of an intervention able to arrest disease progression (1.3-point difference in NSAA change) would take approximately 15 patients per study arm, or larger numbers for smaller effects (Fig. 5).

### Cardiomyopathy

The frequency of DCM was 61% (35/69 patients) overall, higher in the “del 45-x” (17/28, 61%) and “other” (12/21, 57%) than “del x-51” (4/10, 40%) and “del 48” (2/10, 20%) mutation groups. Dystrophin quantity assessed by immunoblot in 30 patients who developed DCM was lower, compared to 22 patients who did not (50% ± 30% vs. 73% ± 29% of control, Mann-Whitney U-test p = 0.009) (Table 4). Measures of LVEF and LVEDV within 1 year before or after baseline evaluation were available for 56 patients (81%). Average LVEF was 56.7 ± 8.9%, while average LVEDV was 63.2 ± 17.7 mL/m^2^, with no significant differences between groups. Frequencies of cardiomyopathy in the whole cohort and in defined mutation groups, as well as average LVEF and LVEDV in the same groups, are detailed in Table 4. There was a trend towards a positive correlation of LVEF (the strongest indicator of overall heart function) with distance covered in the 6MWT (ρ = 0.253, p = 0.06, Supplementary Fig. 2). Accordingly, average baseline 6MWT distance in 34 ambulatory patients affected with DCM was lower (356 ± 182 m) than in 34 DCM-free patients (426 ± 113 m, t-test p = 0.06). These trends could be due either to DCM hindering 6MWT performance *per se*, or to association of DCM to more severe muscle weakness, or both. In order to test these hypotheses, we devised an ANCOVA model with 6MWT distance as dependent variable, and two independent predictors: the presence of DCM, and NSAA score (an indicator of ambulatory muscle weakness, not influenced by fatigability). While the NSAA score remained strongly correlated with 6MWT outcome (p < 0.0001), the presence of DCM showed no significant effect on 6MWT distance in this model, suggesting that cardiomyopathy did not hinder 6MWT performance in this population. This is in keeping with our clinical observation that no patients complained of dyspnea, dizziness, excessive fatigue, or other cardiac symptoms during the 6MWT.

## Discussion

Our study population recapitulates typical genetic and clinical features of BMD: mutations consisting mostly (81%) of single- or multi-exon deletion, and strikingly variable phenotypes^2^,^3^. In “x-51” deletions, immunoblot showed average dystrophin levels of 80%, in the same range as measured by other authors with quantitative immunohistochemistry^9^. While immunoblot and immunohistochemistry are not directly comparable^28^, this finding points to a close-to-normal range of dystrophin abundance in this group, associated with substantially normal strength and function, and no progression over one year. The “del 48” group was clinically similar, despite a lower average amount of dystrophin (67%). These groups presented no loss of the ability to walk or run, an observation somewhat limited by younger age. On the other hand, specific analyses showed that the effect of mutation group on muscle function in our BMD population was independent from age.

The “del 45-x” group, conversely, was confirmed to display “typical” BMD^11^,^12^,^13^, i.e. progressive muscle weakness and wasting, and impaired function. Dystrophin amount averaged 54%, again in the same range as previously shown by other authors using quantitative immunohistochemistry^12^. The exception was represented by the 45–55 deletion, associated with ~90% dystrophin and a mild phenotype, as previously reported^29^. Similar to “x-51” deletions, the absence of the hinge III domain from the internally deleted protein may stabilize dystrophin in patients with del 45–55^8^.

Therefore, as previously proposed by others, we expect skipping of exon 51 to be more effective than skipping of exon 45 in DMD^9^,^12^. Theoretically, AON mixtures promoting multi-exon skipping may be more effective than single-exon skipping, if exons 50–51 were excluded from the resulting internally deleted dystrophin protein. Multi-exon 45–55 skipping has been proposed as a potential treatment for 40–45% DMD patients, although issues with low efficacy and toxicity of AON mixtures hinders the translation of this approach to human patients^30^.

Furthermore, the observation that exon 45–51 and 45–55 deletions have mild or asymptomatic phenotypes suggests that “typically” severe BMD patients with 45–47, 45–48, and 45–49 deletions might also benefit from multi-exon skipping up to exon 51 or 55. On the other hand, inefficient multi-exon skipping could lead to out-of-frame transcripts which would be degraded through nonsense-mediated decay, with the risk of diminishing overall dystrophin levels. Therefore, such an approach remains highly speculative and *in vitro* proof-of-principle studies are warranted.

Some individual patients offered interesting insights into genoype-phenotype correlations. A 13-year-old patient with an exon 3–9 deletion, ablating most of the N-terminal actin binding domain, nevertheless presented a mild phenotype (NSAA 34/34), suggesting functional rescue through secondary actin-binding sites^31^.

The novel missense mutation p.Thr160Pro (exon 6) was associated to “typical” BMD (LoA at 38 years), the mutant proline probably disrupting secondary structures within the actin-binding domain.

A synonym nucleotide substitution (c.4299G > T, p.Gly1433Gly), likely disrupting an ESE in exon 31^27^, caused mild dystrophinopathy (NSAA 34/34) in two brothers aged 35 and 47, with 10~30% dystrophin with slightly reduced molecular weight at immunoblot. These cases represent a rare pathogenetic mechanism, and displayed well-preserved strength despite relatively low dystrophin.

One patient carrying the nonsense mutation c.4980G > A, p.Trp1660* (exon 35) presented relatively mild BMD (age 32, NSAA 32/34) with 29% dystrophin, while another, carrying the mutation c.3843G > A, p.Trp1281* (exon 28), displayed substantial weakness (age 25, NSAA 12/34) with 17% dystrophin. A BMD phenotype is sometimes observed with nonsense mutations within in-frame exons with low splice acceptor site strength and ESE density, leading to endogenous exon skipping^32^. Interestingly, exons 28 and 35 are actually predicted to have relatively strong splice acceptor sites and average-to-high ESE density^32^. Other unidentified factors may dictate alternative splicing in these previously unreported mutations.

Two brothers had an out-of-frame microdeletion in exon 74 (c.10507_10508delAG, p.Lys3505AlaFsX8), and were included because of reduced IHC signal with the anti-rod DYS-1 antibody. One brother lost ambulation at the age of 22, and one is ambulatory at 17. The distal situation of this frameshifting mutation may determine a partial escape of the transcript from nonsense-mediated decay. Unfortunately, no muscle tissue was available for immunoblot.

Two patients with duplications (dup 13–42 and 19–41) and abnormally large dystrophin (estimated around 550 kDa), presented symptomatic BMD. The larger duplication (13–42) was previously described as causing a mild phenotype^33^, but 25 years from the original report the patient’s weakness has progressed (NSAA 12/34 at 35). Conversely, the patient carrying dup 19–41 is more mildly affected (NSAA 33/34 at 47). Sheer protein size, or subverted phasing of protein domains may be dictating phenotype in these patients.

As proposed previously^3^,^9^,^13^,^14^,^15^, we did identify a moderate correlation of dystrophin quantity with phenotype severity, arguably due to molecular mechanisms stabilizing internally deleted *DMD* transcripts and/or dystrophin proteins. We did not observe a clear correlation between dystrophin quantity and phenotype within the most frequent mutation groups (see the “del 45-x” data series in Fig. 2), similar to findings in a Dutch cohort^13^. Correlation within a homogeneous mutation group would reduce confounding effects due to different mutations, and suggest that disease severity be actually dictated by inter-individual variability in dystrophin quantity. Although this analysis is hindered by low patient numbers, challenges in accurate dystrophin quantification^28^, and sampling variability in muscle biopsies, there does not seem to exist such a linear correlation within homogeneous mutation groups. On the other hand, differences in both dystrophin quantity and phenotype severity across different mutation groups, which are consistently observed^3^,^8^,^9^,^11^, suggest that the molecular properties of different internally deleted dystrophin proteins dictate disease severity, by causing varying degrees of downstream pathologic phenomena, such as altered costamere resistance to mechanical stress, membrane hyperpermeability, and disrupted interaction with other proteins (e.g. dystrophin-associated glycoproteins, nitric oxide synthase, syntrophin, and dystrobrevin). It has recently been shown that the inflammatory milieu characterizing active dystrophic pathology, and specifically the activation of TNFα/NF-κB signaling, upregulates several microRNAs targeting the *DMD* transcript, thus inhibiting translation^34^. Therefore, quantitative dystrophin reduction seems a consequence of qualitative dystrophin alterations in BMD, in turn sustaining a vicious cycle of exacerbated pathology. As a corollary, the use of anti-inflammatory agents and NF-κB inhibitors might be beneficial in BMD.

The 6MWT, despite wide variability, was correlated with dystrophin quantity, age, and all other outcomes, supporting its clinical meaningfulness. Mild/asymptomatic patients (NSAA 34/34) always walked more than 400 m, a threshold suggesting low probability of short-term changes in DMD^22^. The exception (6MWT 183 m, NSAA 34/34) was one patient with a hysterical/dystonic gait because of schizophrenia treated with neuroleptics.

The NSAA and TFTs, on the one hand, showed obvious “ceiling effects” in the “del 48” and “del x-51” mutation groups. On the other hand, in “typical” BMD (e.g. “del 45-x”) these measures showed a symmetric distribution, a tight inverse correlation with age (similar to muscle strength scores in the Dutch cohort)^13^, and a strong correlation with 6MWT, suggesting that they are clinically meaningful outcomes.

The 6MWT distance was stable overall after one year. Breakdown of 6MWT changes by mutation revealed apparent “improvements” in a few mildly affected patients, probably due to a “learning effect”. However, a decreasing trend (−12 m) was observed in typical BMD, hinting at a possible detection of significant changes with longer observation, and supporting the clinical meaningfulness of longitudinal 6MWT in BMD.

NSAA changes, although not informative for mild patients (“del 48”, “del x-51”), detected a statistically significant progression (−0.9), driven by a decrease in the “del 45-x” group (−1.3), in itself statistically significant and clinically meaningful, as this decrease relates directly to loss of a motor ability or need for compensation to perform it independently.

These observations have several implications. First, we conclude that functional inclusion criteria (for instance NSAA <32) would be useful when one seeks to measure disease progression in a one-year trial. Second, we provided a preliminary power calculation for a hypothetical BMD trial, which represents a step towards clinical trial readiness. Third, in-depth analyses of loss of function patterns are warranted to improve our understanding of BMD natural history and possibly adapt the DMD-tailored^25^ NSAA scale to BMD. Longitudinal TFT changes did not appear sensitive at one year, and longer observation is probably required to better analyze their behavior in BMD.

For the sake of clarity, we purposely left an in-depth study of DCM onset, outcomes, and genotype-phenotype correlations out of this manuscript, which focuses on skeletal muscle functional measures. Nevertheless, a brief description of DCM frequencies within mutation groups, and a simple comparison of skeletal muscles dystrophin quantity between patients with and without DCM, seem to suggest that more severe skeletal muscle phenotypes are associated with a somewhat higher probability of developing DCM. None of the patients in our cohort showed overt symptoms of heart failure at the time of evaluations. Among outcome measure explored in this study, the 6MWT, which has been originally devised for patient with cardiac and respiratory diseases^35^, and is most heavily influenced by fatigability and exercise intolerance, could be biased by cardiomyopathy. Multivariate analysis including the NSAA (an indicator of ambulation-related muscle weakness, not influenced by fatigability) and the presence of DCM as concurrent predictors of 6MWT performance, seemed to reasonably exclude the presence of such a bias in our population, in keeping with the clinical observation that no patients had cardiac symptoms during the 6MWT. Nevertheless, it is of the utmost importance to remind that if the 6MWT should be adopted as an outcome measure in BMD clinical trials and natural history studies, specific inclusion/exclusion criteria should be devised, based on clinical features such as New York Heart Association classes or echocardiographic parameters, in order to prevent variability due to heart dysfunction from obscuring potential efficacy of a treatment.

We acknowledge several limitations to this study: lack of baseline test/retest, unavailability of immunoblot in all patients, and some loss to longitudinal follow-up. We plan to continue data collection and expand the cohort. Furthermore, the “del 48” and “del x-51” patients were younger, which might bias comparisons to some extent, although older individuals in these groups never showed functional deterioration.

In conclusion, we characterized mutation-specific BMD subphenotypes with longitudinal measures, and predicted better outcomes for exon 51 than 45 skipping in DMD. We showed that only BMD patients with measurable muscle weakness at baseline incur functional deterioration after one year. NSAA and 6MWT hold promise as clinically meaningful outcome measures for BMD trials.

## Additional Information

**How to cite this article**: Bello, L. *et al.* Functional changes in Becker muscular dystrophy: implications for clinical trials in dystrophinopathies. *Sci. Rep.* **6**, 32439; doi: 10.1038/srep32439 (2016).

## Supplementary Material

Supplementary Information

## Figures and Tables

**Table 1:** Age and dystrophin quantity by *DMD* mutation.

Mutation group	Individual mutation	Age (years)			Dystrophin (% of control)		
		n	Mean ± SD	median (range)	n	Mean ± SD	median (range)
“del 45-x”	del 45–47	10	38.1 ± 13.0	40.1 (10.7~55.9)	5	39 ± 27	28 (10~75)
	del 45–48	14	38.5 ± 17.2	38.0 (9.2~69.6)	12	57 ± 28	49 (16~100)
	del 45–49	2	32.1 ± 17.9	32.1 (19.4~44.7)	1	28 ± NA	28 (28~28)
	del 45–55	2	29.3 ± 31.7	29.3 (6.9~51.6)	2	90 ± 14	90 (80~100)
	“del 45-x” total	28	37.2 ± 16.0	38.3 (6.9~69.6)	20	54 ± 29	49 (10~100)
“del 48”	del 48	10	24.2 ± 19.5	14.5 (6.1~65.8)	7	67 ± 21	58 (40~100)
“del x-51”	del 34–51	1	50.7 ± NA	NA	1	70 ± NA	NA
	del 45–51	5	16.8 ± 6.8	16.2 (8.6~25.7)	4	90 ± 12.5	93 (74~100)
	del 48–51	2	37.8 ± 7.2	37.8 (32.7~42.9)	2	64 ± 24	64 (47~81)
	del 50–51	2	14.3 ± 2.1	14.3 (12.9~15.8)	2	82 ± 18	82 (69~95)
	“del x-51” total	10	23.9 ± 14.1	18.7 (8.6~50.7)	9	80 ± 17	81 (47~100)
“other”	del 3–9	1	13.1 ± NA	NA	1	100 ± NA	NA
	rod domain del*	4	33.7 ± 14.2	29 (22.6~54.2)	4	100 ± 0	100 (100~100)
	del 48–49	3	50.2 ± 13.1	54.7 (35.4~60.3)	3	37 ± 38	30 (3~78)
	duplications	2	39.9 ± 6.9	39.9 (35~44.7)	2	30 ± 35	30 (5~55)
	nonsense	2	28.6 ± ± 4.8	28.6 (25.2~32)	2	23 ± 8	23 (17~29)
	missense	1	37.6 ± NA	NA	1	13 ± NA	NA
	small deletions	6	35.4 ± ± 18.1	34.9 (17.4~54.1)	1	39 ± NA	39 (39~39)
	synonym	2	40.7 ± 8	40.7 (35~46.3)	2	23 ± 4	23 (20~26)
	“other” total	21	36.5 ± 14.2	35.0 (16.1~50.3)	16	51 ± 39	34.5 (3~100)
Total		69	33.2 ± 16.5	34.9 (6.1~69.6)	52	59 ± 31	57 (3~100)

SD: standard deviation. del: deletion.

^*^Including deletions of exons 10–25, 10–29, del 11–30.

**Table 2:** Baseline functional measures and functional changes at 1 year by mutation group.

Functional measure	Mutation group	n at baseline	Mean ± SD at baseline	Median (range) at baseline	n with longitudinal data	Mean change ± SD	Median change (range)
6MWT distance (m)	del 45-x	27	347 ± 167	372 (0~615)	27	−12 ± 31§	−15 (−87~45)
	del 48	10	459 ± 121	482 (183~656)	9	14 ± 43.2	79 (−58~79)
	del x-51	10	497 ± 100	474 (386~721)	10	17 ± 71	135 (−90~135)
	other	21	365 ± 147	413 (0~595)	17	4 ± 95	7 (−170~273)
	All BMD	68	391 ± 155	425 (0~721)	54	3 ± 66	−2.5 (−170~273)
NSAA score	del 45-x	27	20.9 ± 11.1	20 (2~34)	20	−1.3 ± 1.7**	−1 (−5~1)
	del 48	10	33.9 ± 0.32	34 (33~34)	9	−0.3 ± 0.5	0 (−1~0)
	del x-51	10	33.7 ± 0.95	34 (31~34)	10	0 ± 0	0 (0~0)
	other	21	23.0 ± 11.2	24 (2~34)	18	−1.3 ± 2.2	0 (−6~1)
	All BMD	68	25.3 ± 10.8	32.5 (2~34)	57	−0.9 ± 1.6***	0 (−6~1)
Run/walk 10 m velocity (m/s)	del 45-x	27	1.55 ± 1.05	1.25 (0~3.33)	18	0.22 ± 0.65	0 (−0.42~2.5)
	del 48	9	3.52 ± 1.01	3.33 (1.67~5)	9	−0.36 ± 0.6	0 (−1.67~0)
	del x-51	10	3.95 ± 1.19	4.17 (2~5)	10	−0.17 ± 0.53	0 (−1.67~0)
	other	21	1.99 ± 1.41	1.67 (0~5)	17	−0.04 ± 0.43	0 (−0.83~0.83)
	All BMD	67	2.31 ± 1.49	3.00 (0~5)	54	−0.03 ± 0.58	0 (−1.67~2.5)
Rise from floor velocity (s^−1^)	del 45-x	28	0.19 ± 0.19	0.17 (0~0.5)	13	0.11 ± 0.25	0 (−0.1~0.8)
	del 48	9	0.52 ± 0.2	0.5 (0.33~1)	9	0.1 ± 0.17	0.5 (0~0.5)
	del x-51	10	0.62 ± 0.28	0.5 (0.33~1)	10	0.01 ± 0.38	0.67 (−0.5~0.67)
	other	21	0.27 ± 0.27	0.17 (0~1)	12	0.024 ± 0.17	0 (−0.17~0.5)
	All BMD	68	0.32 ± 0.28	0.33 (0~1)	44	0.06 ± 0.25	0 (−0.5~0.8)
Climb stairs velocity (steps/s)	del 45-x	28	1.20 ± 1.21	0.8 (0~4)	16	−0.03 ± 0.16	0 (−0.29~0.33)
	del 48	9	2.96 ± 1.25	4 (1.33~4)	9	−0.15 ± 0.73	0 (−2~0.67)
	del x-51	10	3.13 ± 1.14	4 (1.33~4)	10	−0.2 ± 0.71	0 (−2~0.67)
	other	21	1.57 ± 1.38	1.33 (0~4)	16	−0.17 ± 0.76	0 (−2~0.67)
	All BMD	68	1.83 ± 1.46	1.33 (0.00~4.00)	51	−0.13 ± 0.6	0 (−2~0.67)

SD: standard deviation. 6MWT: 6 Minute Walk Test. NSAA: North Star Ambulatory Assessment.

^§^p = 0.059,

^**^p = 0.001.

^***^p < 0.001 (Friedman Rank Sum test).

**Table 3:** Functional changes after 1 year, by dystrophin quantity.

Functional change at 1 year	Dystrophin (% of control)	n	Mean change ± SD	Median change (range)	Correlation between dystrophin % and functional change
6MWT distance (m)	0–33	11	−21 ± 62	−9 (−170~52)	ρ = 0.3, p = 0.055
	34–66	8	4 ± 31	1.5 (−29~51)	
	67–100	23	18 ± 75	10 (−90~273)	
NSAA score	0–33	11	−0.91 ± 1.58	−1 (−5~1)	ρ = 0.025, p = 0.09
	34–66	10	−0.8 ± 1.82	0 (−5~1)	
	67–100	24	−0.38 ± 0.93	0 (−4~0)	
Run/walk 10 m velocity (m/s)	0–33	11	−0.03 ± 0.41	0 (−0.83~0.83)	ρ = 0.22, p = n.s.
	34–66	8	−0.13 ± 0.7	0 (−1.67~0.83)	
	67–100	23	0.09 ± 0.67	0 (−1.67~2.5)	
Rise from floor velocity (s^−1^)	0–33	7	0.16 ± 0.24	0 (0~0.5)	ρ = 0.07, p = n.s.
	34–66	7	−0.01 ± 0.1	0 (−0.17~0.17)	
	67–100	22	0.07 ± 0.32	0 (−0.5~0.8)	
Climb stairs velocity (steps/s)	0–33	10	0 ± 0.26	0 (−0.29~0.67)	ρ = 0.06, p = n.s.
	34–66	9	0.11 ± 0.33	0 (−0.33~0.67)	
	67–100	22	−0.35 ± 0.83	0 (−2~0.67)	

6MWT: 6 Minute Walk Test. NSAA: North Star Ambulatory Assessment.

**Table 4:** Prevalence of cardiomyopathy and echocardiographic parameters by mutation group.

	Prevalence of cardiomyopathy by mutation group									
	del 45-x (n = 28) n%		del 48 (n = 10) n%		del x-51 (n = 10) n%		other (n = 21) n%		total (n = 69) n%	
Dilated cardiomyopathy	17	61%	2	20%	4	40%	12	57%	35	51%
No dilated cardiomyopathy	11	39%	8	80%	6	60%	9	43%	34	49%
Orthotopic heart transplantation*	1	4%	0	0%	0	0%	2	10%	3	4%
	Ecocardiographic parameters (within 1 year before or after baseline functional evaluation)									
	del 45-x (n = 21)†		del 48 (n = 8)†		del x-51 (n = 9)†		other (n = 18)†		total (n = 56)†	
Average LVEF (%) ± SD	54.8 ± 9.7		56.5 ± 4.5		59.7 ± 5.4		57.2 ± 10.6		56.7 ± 8.9	
LVEDV (mL/m^2^) ± SD	64.5 ± 18.8		63.4 ± 7.5		62.0 ± 12.0		62.3 ± 22.6		63.2 ± 17.7	

See Methods for definition of cardiomyopathy.

^*^Patients who had undergone heart transplantation were also included in the “dilated cardiomyopathy” count.

^†^Echocardiographic parameters within 1 year before or after baseline functional evaluation were not available for all patients. LVEF: Left Ventricular Ejection Fraction. LVEDV: Left Ventricular End Diastolic Volume.
