# Supplementary material for: Functional changes in Becker muscular dystrophy: implications for clinical trials in dystrophinopathies
Source: Sci Rep. 2016 Sep 1;6:32439. doi: 10.1038/srep32439 (PMC5007528; doi:10.1038/srep32439)
Supplement: Supplementary Information [file srep32439-s1.pdf]

Supplementary information for the paper:

"Functional changes in Becker muscular dystrophy: implications for clinical trials in dystrophinopathies"

Luca Bello, MD, PhD[1]; Paola Campadello, MD[1]; Andrea Barp, MD[1]; Marina Fanin, PhD[1]; Claudio Semplicini, MD[1]; Gianni Sorarù, MD, PhD[1]; Luca Caumo[1]; Chiara Calore, MD, PhD[2]; Corrado Angelini, MD[1]; Elena Pegoraro, MD, PhD[1]*.

[1]Department of Neurosciences, University of Padova, Padova, Italy

[2]Department of Cardiac, Thoracic and Vascular Sciences, Cardiology Clinic, University of Padova, Padova, Italy

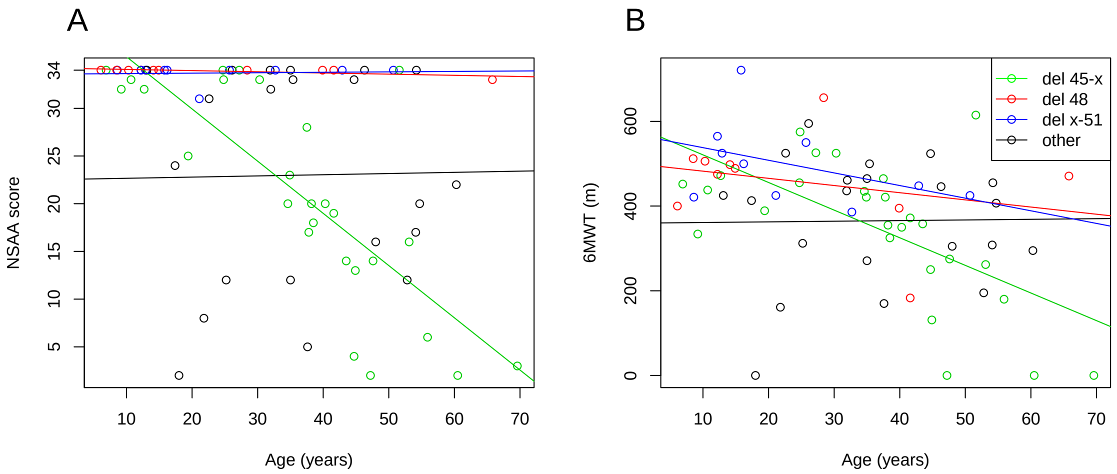

Supplementary Fig. 1. Different slopes of NSAA and 6MWT plotted against age between mutation groups. There is a downward slope of baseline NSAA values plotted against age in the “del 45-x” group, while baseline NSAA appears stable at the high scores for “del 48” and “del x-51” (Panel A). There is a similar behaviour of 6MWT values, with a steeper downward slope in “del 45-x” compared to “del 48” and “del x-51” (Panel B). Values in “other” mutations are more variable (i.e. this group is more heterogeneous and includes both mild and severe patients).

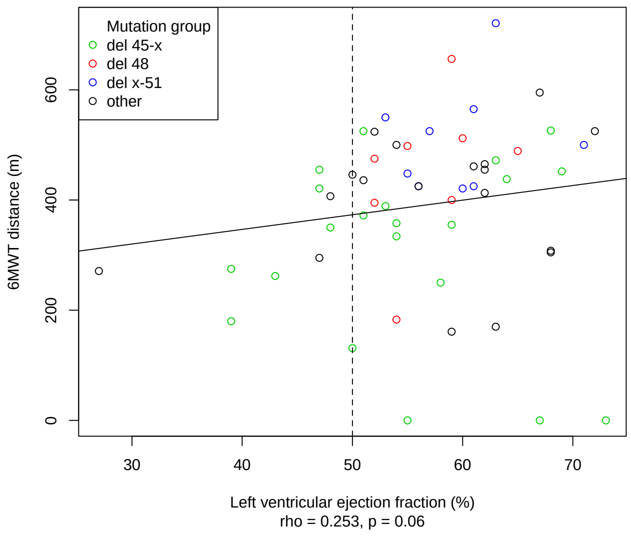

Supplementary Fig. 2. Scatter plots of 6MWT distance and left ventricular ejection fraction. There is a trend towards a positive correlation between 6MWT distance and left ventricular ejection fraction (obtained within 1 year of functional evaluation).
